# Optimization of Environmental Conditions for Microbial Stabilization of Uranium Tailings, and the Microbial Community Response

**DOI:** 10.3389/fmicb.2021.770206

**Published:** 2021-12-13

**Authors:** Ying Lv, Chuiyun Tang, Xingyu Liu, Mingjiang Zhang, Bowei Chen, Xuewu Hu, Susu Chen, Xuezhe Zhu

**Affiliations:** ^1^National Engineering Research Center for Environment-Friendly Metallurgy in Producing Premium Non-ferrous Metals, GRINM Group Co., Ltd., Beijing, China; ^2^School of Metallurgical and Ecological Engineering, University of Science and Technology Beijing, Beijing, China; ^3^GRINM Resources and Environment Technology Co., Ltd., Beijing, China; ^4^General Research Institute for Non-ferrous Metals, Beijing, China; ^5^GRIMAT Engineering Institute Co., Ltd., Beijing, China

**Keywords:** environmental conditions, sulfate-reducing bacteria, phosphate-solubilizing bacteria, microbial community response, microbial stabilization, uranium tailings

## Abstract

Uranium pollution in tailings and its decay products is a global environmental problem. It is of great significance to use economical and efficient technologies to remediate uranium-contaminated soil. In this study, the effects of pH, temperature, and inoculation volume on stabilization efficiency and microbial community response of uranium tailings were investigated by a single-factor batch experiment in the remediation process by mixed sulfate-reducing bacteria (SRB) and phosphate-solubilizing bacteria (PSB, *Pantoea* sp. grinm-12). The results showed that the optimal parameters of microbial stabilization by mixed SRB-PSB were pH of 5.0, temperature of 25°C, and inoculation volume of 10%. Under the optimal conditions, the uranium in uranium tailings presented a tendency to transform from the acid-soluble state to residual state. In addition, the introduction of exogenous SRB-PSB can significantly increase the richness and diversity of endogenous microorganisms, effectively maintain the reductive environment for the microbial stabilization system, and promote the growth of functional microorganisms, such as sulfate-reducing bacteria (*Desulfosporosinus* and *Desulfovibrio*) and iron-reducing bacteria (*Geobacter* and *Sedimentibacter*). Finally, PCoA and CCA analyses showed that temperature and inoculation volume had significant effects on microbial community structure, and the influence order of the three environmental factors is as follows: inoculation volume > temperature > pH. The outcomes of this study provide theoretical support for the control of uranium in uranium-contaminated sites.

## Introduction

With the rapid development of the nuclear power industry, tests of depleted uranium weapons, and other human activities such as uranium mining and metallurgy, the content of uranium and its compounds in the environment has increased significantly (Brugge et al., 2005). Uranium not only is radioactive, which can cause radiation to the human body, but also has strong chemical toxicity (Lu et al., 2018; Selvakumar et al., 2018). In the natural environment, uranium will cause the decrease in cellular activity and metabolic activity of soil microorganisms after entering soil and affect the whole soil ecosphere; once in the body, uranium and its compounds are apt to bind with phosphorylated peptides, causing damage to the kidneys, bones, and brain (Tang et al., 2021). Undoubtedly, the world will face a more serious situation of soil contaminated by uranium in the future with the further development and application of nuclear energy. In particular, as a kind of solid waste produced in the process of uranium extraction, uranium tailings have the characteristics of large yield, complex composition, high toxicity, and difficulty in purification and treatment (Fernandes, 1996). Therefore, it is urgent to develop an efficient and economical uranium pollution remediation technology.

The remediation technology of uranium pollution can be divided into physical, chemical, and biological methods in principle. Among them, physical–chemical technologies have the advantages of high remediation efficiency in single treatment (Wen et al., 2018), but they are mainly targeted at sites with small area and high pollution degree, and they have problems such as complex operation process, high treatment cost, and secondary pollution, which limit the application scope of these methods to a certain extent (Shen et al., 2019; Woods, 2019; Zhang et al., 2019). However, bioremediation is exactly suitable for uranium-contaminated sites with large polluted area and low pollution degree and has received great attention in recent years (Jing et al., 2020), wherein microbial remediation technology has the outstanding advantages of low cost, easy operation, and small disturbance to the environment, providing a greener, economic, and stable way for the remediation of uranium-contaminated sites and is considered to have great research value and broad application prospects (Lakaniemi et al., 2019). In the field of uranium pollution control, many studies have shown that sulfate-reducing bacteria (Hu et al., 2011; He et al., 2021) and phosphate-solubilizing bacteria (Zhong et al., 2021) can significantly alleviate the toxicity of uranium in the environment through bio-reduction (Zhong et al., 2019), bio-precipitation (Yao, 2017), bio-adsorption (Yong and Macaskie, 2010), and other approaches. However, there is limited information on the combined effect of the two functional strains.

At present, research on the control treatment of uranium pollution has developed from efficient removal to stabilization, but it is mostly focused on the treatment of uranium pollution in water and soil (Kim et al., 2013; Khan, 2020; Zhong et al., 2021), and there are few reports about the stabilization of tailings. Many studies have shown that pH, temperature, inoculation volume, and other environmental factors can change the functional expression of microorganisms by affecting their growth and metabolism (Ji and Wei, 2006; Senko et al., 2007; Zhao, 2014). Therefore, it is necessary to determine the most suitable parameters for the growth of functional microorganisms through experiments, so as to enhance the stabilization efficiency (Liu, 2019). In addition, it is necessary not only to study the stabilization effect of microorganisms on uranium but also to consider the response of the microbial community during the remediation process, due to the complex composition of tailings and the variously endogenous microbial community, so as to achieve the stability and functional control of the microbial community. The microbial community structure has always been the focus of microbial ecology and environmental studies, which determines the characteristics and strength of ecological functions (Wang et al., 2019). Therefore, it can provide a reliable basis for optimizing the community structure, regulating the community function, and discovering new important microbial functional groups by analyzing the dynamic changes of the microbial succession.

In this study, a strain of laboratory-preserved phosphate-solubilizing bacteria (*Pantoea* sp. grinm-12) were co-cultured with SRB, then the effects of environmental conditions (temperature, pH, and inoculation volume) on uranium tailings during the microbial stabilization process were investigated through a single-factor batch experiment. The specific objectives of this study were to investigate (1) the changes of pH and Eh of uranium tailings under different environmental conditions, in order to understand the basic situation of the reaction system; (2) the chemical form changes of uranium under different environmental conditions, so as to probe into the migration and transformation of uranium; and (3) the succession and response of the microbial community during the microbial stabilization process by high-throughput sequencing. Finally, the optimal environmental parameters for *in situ* stabilization of uranium tailings were determined by combining the above outcomes.

## Materials and Methods

### Materials

The uranium tailings used in this experiment were collected from a uranium tailings repository located in Fuzhou city, Jiangxi Province, southeast of China. Moreover, the chemical properties of the sampled tailings were shown in our previous study (Tang et al., 2021), as U, Mn, Fe, Ca, and SO_4_^2–^ were the main compositions. The sulfate-reducing bacteria and phosphate-solubilizing bacteria used in this study were preserved in the National Engineering Laboratory of Biohydrometallurgy, China. Among them, the dominant functional strain of the sulfate-reducing bacteria is *Desulfovibrio* (with strain number of *Desulfovibrio vulgaris EM2*), and the phosphate-solubilizing bacteria are identified as *Pantoea* sp. The components of the mixed culture medium are as follows: 1.2 g l^–1^ glucose, 1.5 g l^–1^ NH_4_NO_3_, 0.5 g l^–1^ NaCl, 1 g l^–1^ MgSO_4_, 0.5 g l^–1^ (NH_4_)_2_SO_4_, 1 g l^–1^ MgCl_2_, 2.5 g l^–1^ Ca_3_(PO_4_)_2_ (providing phosphorus source for PSB), 0.2 g l^–1^ KCl, 0.5 g l^–1^ C_3_H_5_O_3_Na, and 0.5 g l^–1^ Na_2_SO_4_.

### Microbial Stabilization of Uranium Tailings by Sulfate-Reducing Bacteria-Phosphate-Solubilizing Bacteria

The radioactive sampling method was used to take samples around the open-area uranium tailings repository, and all samples were then mixed evenly to make them representative. Then, the stabilization experiments were carried out in conical flasks with a solid–liquid (uranium tailings-medium) ratio of 1:1. Considering the factors that can be controlled in engineering practice, three variables including pH, temperature, and inoculation volume were selected for single-factor batch experiments in this study. In view of these three environmental factors, different gradients were set respectively, to investigate the pH, Eh, chemical form transformation of uranium, and response of the microbial community in the bioremediation process. The specific parameters of experimental settings are shown in Table 1, and the optimal environmental conditions were finally determined one by one. All experimental groups were repeated three times, and the final values of pH, Eh, and chemical form were the average outcomes of the parallel groups, while the microbial response analysis was based on high-throughput sequencing results of a representative sample under the same conditions.

**TABLE 1:** Parameter setting in microbial stabilization experiments.

Sample	Solid–liquid ratio	Initial pH	Temperature (°C)	Inoculation volume* (%)
1	1:1	3	25	10
2	1:1	5	25	10
3	1:1	7	25	10
4	1:1	5	15	10
5	1:1	5	25	10
6	1:1	5	35	10
7	1:1	5	25	5
8	1:1	5	25	10
9	1:1	5	25	20

*The mixed bacterial solution used in this study was obtained by mixing SRB and PSB at a volume ratio of 1:1 after activization of culture (bacterial concentration was approximately 1 × 10^7^ cells⋅ml^–1^), under the conditions of 25°C and oscillating at 30°C for 3 days.

### Measurements

The pH of the leaching solution was determined by a glass electrode, (Orion 3 Star pH Benchtop, Thermo Scientific, Waltham, MA, United States). The Eh measurement was carried out by an ORP controller (PC-350, Suntex, China). The chemical form of uranium was detected by the BCR continuous extraction method (Zhang et al., 2012). High-throughput sequencing was assisted by Majorbio (Shanghai, China).

## Results and Discussion

### Effect of pH on Microbial Stabilization Process and Microbial Community Response

#### pH and Eh Changes During the Microbial Stabilization Process

**FIGURE 1:**
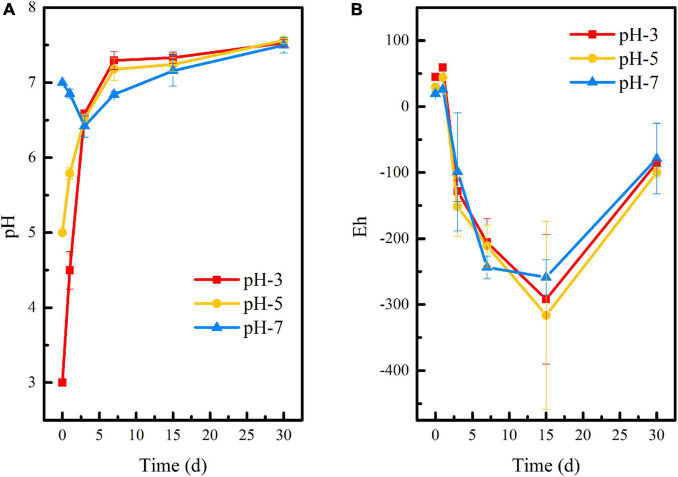
Changes of **(A)** pH and **(B)** Eh during the microbial stabilization process of SRB-PSB under different pH.

Figures 1A,B showed that the initial pH of the medium has no significant effect on the pH and Eh of the stabilization system, and the overall change trend of the three groups remained consistent throughout the 60-day reaction period. The difference was that when the initial pH adjusted to 3 or 5, the pH of the system increased rapidly within 0–7 days, reaching 7.29 and 7.17, respectively. With the extension of time, the pH of the environmental system gradually stabilized to neutral. When the initial pH was 7, the pH of the system decreased slightly in the first 3 days and then gradually stabilized in the neutral range, too. This is due to the fact that a large amount of lime was added to the uranium tailings before it was stockpiled, thus acting as a buffer to the pH of the stabilization system. It is shown in Figure 1B that Eh showed a downward trend at the initial remediation stage and reached the lowest on the 15th day, which was −292, −316, and −258 mV in pH-3, pH-5, and pH-7, respectively. However, Eh showed a rising process after stabilization for 15 days. This may be due to the lack of nutrients in the culture system at the later stage, which leads to inhibition of the metabolic activity of SRB and difficulty in maintaining the reducing atmosphere in the environmental system.

#### Chemical Form Transformation During the Microbial Stabilization Process

**FIGURE 2:**
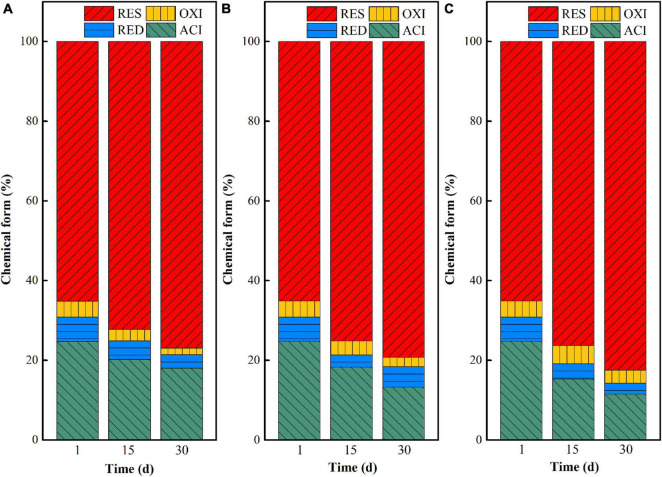
Chemical form (ACI—acid-soluble state; RED—reducible state; OXI—oxidizable state; RES—residual state) changes of uranium in uranium tailings under different pH, **(A)** pH-3; **(B)** pH-5; **(C)** pH-7.

The chemical form changes of uranium in uranium tailings under different pH are shown in Figure 2. At the beginning stage (at day 1), uranium mainly existed in the form of residual and acid-soluble states, as the total content exceeded 90%. With the extension of remediation time, the proportion of acid-soluble uranium decreased and the residual uranium increased in the three groups, indicating that the three systems all could effectively stabilize uranium in the uranium tailings and achieve *in situ* stabilization of uranium. Among them, the transformation ratio of uranium from the acid-soluble state to residual state in the pH 7 group was the largest, followed by the remediation system with pH = 5. That is, the closer the pH is to the neutral condition, the more favorable the chemical form of uranium is to transform from the acid-soluble state to residual state. Specifically, the uranium in the acid-soluble state decreased by 6.71, 11.53, and 13.25% in pH 3, pH 5, and pH 7 after 30 days of microbial stabilization, respectively, and the residual uranium increased by 11.82, 14.20, and 17.83%, respectively. This may be because the growth and functional expression of the mixed microorganisms are restricted at lower pH. When the pH of the system is close to neutral, the microbial activity is higher under the premise that all other environmental conditions are consistent, thus increasing the higher stabilization rate of uranium. In addition, the control group (aseptic inoculation experiments) showed that the unstable uranium remained at a relatively high level after the leaching effect of medium in our previous study (Tang et al., 2021). Therefore, it can be inferred that uranium conversion is mainly based on microbial metabolism.

#### Microbial Community Response

The information of microbial succession is an important aspect of marking environment change. The α diversity index is often used to describe the richness and diversity of microbial communities (Chen et al., 2018). As is shown in Table 2, α diversity indexes under different pH of medium were different, and the variation of ACE and Chao 1 indexes generally showed a trend of decreasing at first and then increasing. However, ACE and Chao 1 of the three reaction systems all reached their peak values on day 30; that is, the microbial richness reached its maximum. This indicated that exogenous microbial stimulation (SRB-PSB) caused the fluctuation of the endogenous microbial community structure at the initial stage of reaction. Then, the adaptability of exogenous microorganisms to the local environment might be enhanced with the extension of reaction time, resulting in the improvement of microbial richness. This indicated that the stimulation of the SRB-PSB coculture system in this study was conducive to increasing local microbial abundance and realizing the reconstruction of the *in situ* microbial system.

**TABLE 2:** Variation of α diversity indexes under different pH.

Group*	Shannon	Simpson	ACE	Chao 1	Coverage
pH-3-1	0.95	0.66	112.01	113	0.999
pH-3-3	1.77	0.27	195.59	163	0.999
pH-3-7	1.40	0.42	114.89	105	0.999
pH-3-15	2.57	0.19	186.78	186.4	0.999
pH-3-30	2.85	0.13	194.51	192.81	0.999
pH-5-1	1.60	0.34	192.33	182.89	0.999
pH-5-3	1.54	0.33	70.40	70	0.999
pH-5-7	1.58	0.38	78.70	78	0.999
pH-5-15	2.79	0.12	192.28	191.94	0.999
pH-5-30	2.57	0.19	193.55	192.57	0.999
pH-7-1	1.44	0.36	131.42	109	0.999
pH-7-3	1.67	0.29	60.40	60.17	0.999
pH-7-7	1.75	0.28	79.02	84.5	0.999
pH-7-15	2.59	0.17	156.73	156.83	0.999
pH-7-30	1.81	0.31	167.62	165.07	0.999

*Remediation systems with different initial pH were sampled at days 1, 3, 7, 15, and 30 and named according to pH and sampling time. For example, the 5 samples in pH-3 were labeled pH-3-1, pH-3-3, pH-3-7, pH-3-15, and pH-3-30, respectively.

The Shannon index and Simpson index in the pH-3 group showed a trend of fluctuation: the Shannon index decreased after an increase, but then increased again, while the Simpson index changed on the contrary. The Shannon index in pH-5 decreased firstly and then increased with the extension of remediation time, while the Simpson index showed no special change rule. The Shannon index in pH-7 showed a trend of first increase and then decrease; correspondingly, the Simpson index showed a trend of first decrease and then increase. Considering that microbial diversity is positively proportional to the Shannon index and inversely proportional to the Simpson index (Chen et al., 2018), it can be inferred that the introduction of exogenous microorganisms also affected the local microbial diversity. However, the Shannon index reached the peak and the Simpson index reached the valley at different times in the group with different initial pH. When the initial pH of medium was 5 and 7, the Shannon and Simpson indexes reached the maximum value at day 15, which were different from that of the pH-3 group (reached the peak value at day 30). That is, the change in microbial diversity in the pH-3 group lagged behind that in the pH-5 and pH-7 groups. It may be because the growth of microorganisms in the system is inhibited when the initial pH of the medium is too low. As the reaction progressed, the pH of the system gradually tended to be neutral for microbial growth (Figure 1A), and microorganisms can proliferate in this favorable environment. On the 30th day, compared with the samples at day 15, the Shannon and Simpson indexes in the pH-5 and pH-7 groups showed decreasing and increasing trends, respectively, which may be due to the decrease in microbial diversity caused by the lack of nutrients in the later stage.

**FIGURE 3:**
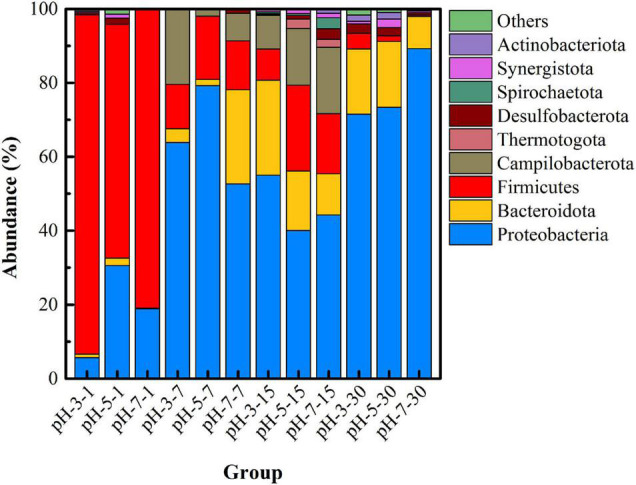
Microbial community structure at the phylum level under different pH.

In order to further understand the changes in microbial community structure under different pH, high-throughput sequencing was used to reveal the succession of the dominant microbial community versus time. Figure 3 shows the microbial community structure at the phylum level with abundance greater than 1% in each group under different pH. Firmicutes, Proteobacteria, Bacteroidota, Desulfobacterota, and Synergustota were mainly detected in the initial remediation system, and their cumulative abundance exceeded 99%. Among them, Firmicutes is the main phylum of microorganisms in uranium-contaminated sites, which have been reported in many literatures (Suriya et al., 2017), and Desulfobacterota is a type of SRB that is widely recognized as having the function of sulfate-reducing capacity (Chen et al., 2019). Specially, at day 7, the abundance of Campilobacterota (20.31%) in the pH-3 group was much higher than that in the other two groups, and the abundance of Bacteroidota in the pH-7 group was the highest (25.45%). On the 15th day of the remediation, the main microorganisms at the phylum level were Proteobacteria, Bacteroidota, Firmicutes, Desulfobacterota, Synergustota, Spirochaetota, and Actinobacteriota. What is noteworthy is that, when the remediation time was 15 days, the abundance of Firmicutes, Campilobacterota, Desulfobacterota, and Synergustota in pH-5 and pH-7 groups was much higher than that in the pH-3 group. As the first three microorganisms happen to be the functional microorganisms related to the remediation of heavy metal pollution (Sharp et al., 2011; Carrier et al., 2020), they thus play an important role in the stabilization process.

The community composition was intuitively studied by heatmap analysis at the genus level of the samples after remediation for 15 days; the results are shown in Supplementary Figure 1. Firstly, *Azospirillum*, *Enterobacteriaceae*, *Sulfurospirillum*, *Proteiniphilum*, *Macellibacteroides*, *Pseudomonas*, *Bacteroides*, *Desulfosporosinus*, *Desulfovibrio*, and *Sphaerochaeta* were observed in all groups. Considering that the high stability of the microbial community structure is an important factor to realize ecological function (Li et al., 2019), it can be concluded that the introduction of exogenous functional microorganisms (SRB-PSB) will not destroy the *in situ* ecological stability and can coexist with endogenous microorganisms in this study. Among the various microorganisms, *Azospirillum* is a common nitrate-reducing bacteria (Caiubi et al., 2020), and nitrate has been regarded as a critical substance that determines the prevailing redox conditions in the environment, and in turn the behavior of Uranium (Bonotto et al., 2019); *Desulfosporosinus* and *Desulfovibrio* are typical sulfate-reducing bacteria that can adapt to a uranium-polluted environment and facilitate the stabilization of uranium pollution (Suzuki et al., 2004); in addition, some studies have also proved that uranium pollution in the environment can be removed by *Pseudomonas* through biological mineralization (Choudhary and Sar, 2011). Compared with the three groups under different pH, it can be concluded that the effects of initial pH had significant differences in the reconstruction of the microbial community. For example, in the pH-5 group, the abundance of *Planococcacese*, *AUTHM297*, and *Pseudoxanthomaonas* was much higher than that in pH-3 and pH-7 groups. Among them, *Planococcacese* and *Pseudoxanthomaonas* are microorganisms with biological mineralization function, which is beneficial to uranium removal and stabilization (Chuiyun et al., 2021). In addition, the sulfate-reducing bacteria (*Desulfosporosinus* and *Desulfovibrio*) and iron-reducing bacteria (*Geobacter* and *Stenotrophomonas*) in pH-7 and pH-5 groups were significantly more abundant than those in the pH-3 group. This may be due to the construction of a more suitable reductive microbial stabilization system under neutral conditions.

Based on the above analysis, the remediation system of the pH-5 group can significantly increase microbial richness and diversity and effectively maintain the reductive system (Figure 3 and Supplementary Figure 1), which can promote the construction of functional flora. In addition, the pH-5 group can significantly reduce the Eh of the environmental system (maintaining a reductive environment) (Figure 1B) and can greatly promote the trend of transforming acid-soluble uranium to residual uranium (Figure 2). Therefore, the medium with an initial pH of 5 was finally selected for the subsequent microbial stabilization process.

### Effect of Temperature on Microbial Stabilization Process and Microbial Community Response

#### pH and Eh Changes During the Microbial Stabilization Process

**FIGURE 4:**
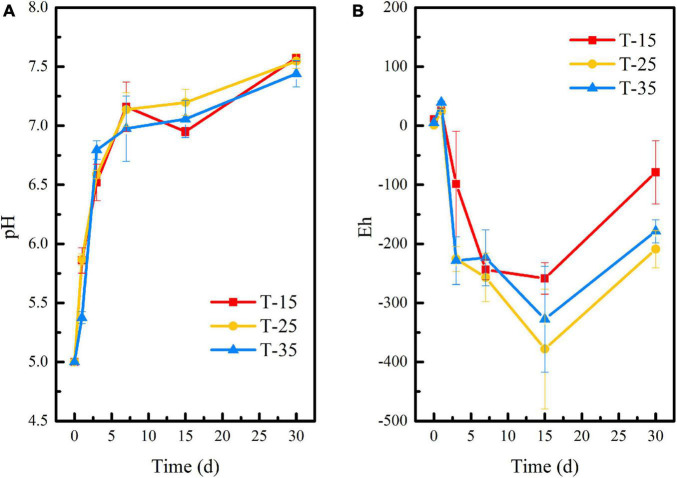
Changes of **(A)** pH and **(B)** Eh during the microbial stabilization process of SRB-PSB under different temperatures.

Figure 4 shows that temperature had a slight effect on pH and Eh of the remediation system, but there was no significant difference among each group. Especially in the first 7 days of reaction, the pH curves at 15°C and 25°C were almost the same. The Eh of T-25 and T-35 groups was basically identical in the first 3 days. At the later stage of reaction, the pH and Eh of each system gradually appeared different. Interestingly, although the Eh in all three groups showed an upward trend from the 15th day to the 30th day, it kept the order of T-15 > T-35 > T-25 all along. Specifically, the pH of the remediation system at different temperatures rose rapidly within 0–7 days and reached 7.16 (T-15), 7.14 (T-25), and 6.98 (T-35), respectively, at day 7 and gradually stabilized at neutral with the extension of time. Within 15 days of the initial reaction, the Eh of the remediation system decreased significantly and reached the lowest values at day 15, which were −258.67 mV (T-15), −378 mV (T-25), and −327.67 mV (T-35), respectively, which were conducive to the growth and functional expression of reducing microorganisms in the remediation system. This in turn promoted the stabilization of uranium. Similar to that shown in Figure 1B, Eh showed an upward trend in the later stage of reaction, that is, the reductive environment was destroyed. However, the Eh at day 30 in T-25 and T-35 groups was significantly lower than that in the T-15 group, indicating that the conditions of 25 and 35°C were more conducive to the maintenance of the reductive system and the subsequent stabilization reaction of reducing bacteria to uranium.

#### Chemical Form Transformation During the Microbial Stabilization Process

**FIGURE 5:**
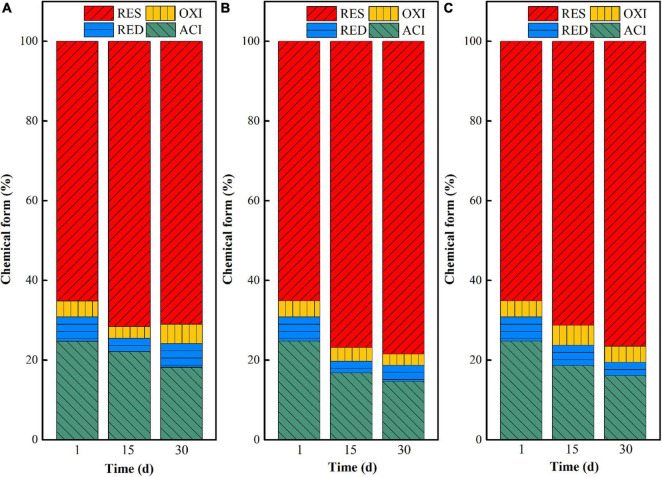
Chemical form changes of uranium in uranium tailings under different temperatures: **(A)** T-15; **(B)** T-25; an d **(C)** T-35.

Temperature has an important influence on the chemical form transformation of uranium in the *in situ* microbial stabilization process, because the growth, metabolism, and enzyme activities of microorganisms will be inhibited at too low or too high temperature (Liu et al., 2018; Lv et al., 2021). In this study, the chemical forms of uranium in uranium tailings reacted with the mixed SRB-PSB system for 1, 15, and 30 days at temperatures of 15, 25, and 35°C, respectively, were compared. The results are shown in Figure 5. Overall, the chemical forms of uranium changed from the acid-soluble state to residual state with the extension of reaction time under the same temperature, indicating that SRB-PSB can effectively achieve the stabilization of uranium. To be specific, when the temperature was set at 15°C, the uranium in the acid-soluble state decreased from 24.75 to 22.17% on the 15th day, and the uranium in residual state increased from 65.14 to 71.55%; however, a small amount of residual uranium appeared to be re-dissolved on the 30th day. Under the temperature of 25°C, the chemical forms of uranium changed significantly in the first 15 days: the content of uranium in the acid-soluble state decreased by 9.95%, and the residual state increased by 13.37%, which was much higher than that in the T-15 group. Moreover, with the extension of time, the stabilization effect is continuously enhanced. When the temperature was 35°C, a similar trend was observed: the content of uranium in the acid-soluble state decreased by 8.6%, and that in the residual state increased by 11.43%, which was slightly lower than that in the T-25 group.

#### Microbial Community Response

As is shown in Table 3, there were significant differences in α diversity index at different temperatures. In the T-15 group, the changes in ACE and Chao 1 indexes basically showed a trend of first decreasing and then increasing and reached the peak at day 30, with maximum values of 131.06 and 137.6, respectively. In the T-25 group, ACE and Chao 1 showed a trend of decreasing at first followed by continuous increasing and finally reaching the maximum values on day 30 with values of 248.81 and 248.25, respectively. However, ACE and Chao 1 in the T-35 group reached the peak values at day 15, with values of 196.11 and 200, respectively. A comparative analysis of maximum values of the ACE and Chao 1 indexes under different temperatures showed that T-25 > T-35 > T-15. This may be because when the temperature is too low, the growth of microorganisms is inhibited, resulting in low bacterial abundance; the reason why the values in the T-35 group were lower than those in the T-25 group is likely the rapid growth of endogenous microorganisms during the remediation process, which leads to the rapid consumption of nutrients in the environment, thus reducing the microbial richness in the later stage. In the T-15 and T-25 groups, the Shannon index reached the peak values at day 30, which were 2.03 and 3.40, respectively, while the Shannon index in the T-35 group reached the maximum value of 3.19 at day 15. The valley values of the Simpson index were obtained on the 30th day in the T-15 group, while in T-25 and T-35 the valley values were obtained when the remediation times were 15 and 30 days, respectively. Compared with the maximum values of the Shannon index, the microbial diversity was as follows: T-25 > T-35 > T-15. This indicated that when the temperature was relatively high (25 and 35°C), it was beneficial to increase microbial diversity.

**TABLE 3:** Variation of α diversity indexes under different temperatures.

Group*	Shannon	Simpson	ACE	Chao 1	Coverage
T-15-1	1.82	0.26	152.07	144.18	0.999
T-15-3	1.90	0.21	70.19	65.1	0.999
T-15-7	1.39	0.45	72.08	72.33	0.999
T-15-15	1.87	0.24	79.19	73.5	0.999
T-15-30	2.03	0.30	131.06	137.6	0.999
T-25-1	1.15	0.47	88.68	94.14	0.999
T-25-3	1.55	0.33	73.17	82	0.999
T-25-7	2.33	0.17	105.36	107.14	0.999
T-25-15	2.62	0.14	161.42	161.77	0.999
T-25-30	3.40	0.07	248.81	248.25	0.999
T-35-1	1.73	0.28	119.92	116.17	0.999
T-35-3	1.95	0.24	78.08	75.55	0.999
T-35-7	2.80	0.09	113.30	111.6	0.999
T-35-15	3.19	0.08	196.11	200	0.999
T-35-30	2.44	0.14	146.61	146.4	0.999

*Remediation systems with different temperatures were sampled at days 1, 3, 7, 15, and 30 and named according to temperature and sampling time. For example, the 5 samples in T-15 were labeled T-15-1, T-15-3, T-15-7, T-15-15, and T-15-30, respectively.

**FIGURE 6:**
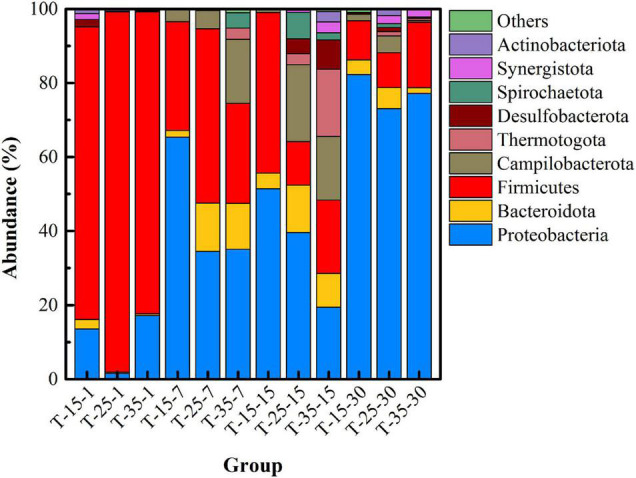
Microbial community structure at the phylum level under different temperatures.

Figure 6 shows the succession of the microbial community structure at the phylum level under different temperatures. On the whole, the dominant microbial species in uranium tailings of the same reaction period were highly consistent, but the proportion was significantly different. Specifically, microorganisms at the phylum level mainly included Firmicutes, Proteobacteria, Bacteroidota, Desulfobacterota, and Synergustota at the initial stage of remediation, which were consistent with the results shown in Figure 3. In the T-25 group, the abundance of Firmicutes was higher than the other two groups at first and 7th days. On the 7th day of the remediation, the abundance of Firmicutes and Proteobacteria changed most dramatically, and the abundance of Bacteroidota and Campilobacterota in the remediation system also increased gradually with the increase in temperature, far exceeding that of the T-15 group. At day 15, the microbial diversity in the T-25 and T-35 groups was much higher than that in the T-15 group, and the microorganisms mainly included Firmicutes, Proteobacteria, Bacteroidota, Desulfobacterota, Synergustota, Thermotogota, and Actinobacteriota. Then the dominant microorganisms in the three groups were basically the same at day 30; in particular, Desulfobacterota, was most abundant in the T-25 group.

A heatmap of the microbial community at the genus level after microbial stabilization for 15 days at different temperatures is shown in Supplementary Figure 2. First of all, *Enterobacteriaceae*, *Sulfurospirillum*, *Proteiniphilum*, *Azospirillum*, *Macellibacteroides*, *Pseudomonas*, *Bacteroides*, and *Desulfosporosinus* were all observed in the three groups. The functions of *Azospirillum*, *Desulfosporosinus*, and *Desulfovibrio* have been described previously. Therefore, the high abundance of *Desulfosporosinus* detected in the T-15 group indicated that the T-15 group could also achieve uranium stabilization to a certain extent. In addition, in the T-25 and T-35 groups, the abundance of *Desulfurispora*, *Desulfofarcimen*, *Petriniphilum*, *Desulfovibrio*, and *Geobacter* was much higher than that in the T-15 group. As these microorganisms have the ability of metal reduction and sulfate reduction, they have greater remediation potential for uranium stabilization. Combined with Figure 5 and Supplementary Figure 1, it was found that the temperature of 25°C was more conducive to the stabilization of uranium and the functional succession of the microbial community.

### Effect of Inoculation Volume on the Microbial Stabilization Process and Microbial Community Response

#### pH and Eh Changes During the Microbial Stabilization Process

**FIGURE 7:**
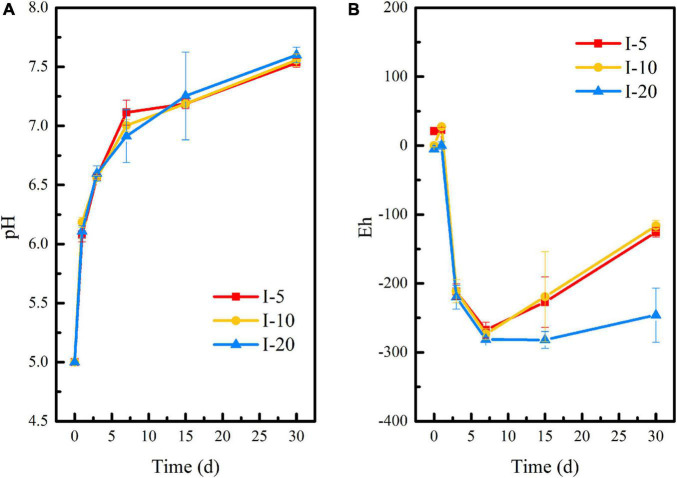
Changes of **(A)** pH and **(B)** Eh during the microbial stabilization process of SRB-PSB under different inoculation volumes.

The changes in pH and Eh of uranium tailings under different inoculation volumes are shown in Figure 7. On the whole, the pH of I-5, I-10, and I-20 groups had the same change trend. Combined with Figures 1A, 4A, this was attributed to the buffering effect of residual lime in uranium tailings. Compared with the reaction system with different pH and temperatures, the pH of each group showed a continuous rise in the period of 0–30 days under different inoculation volumes, and the above platform (shown in Figures 1A, 4A) did not appear. Furthermore, the pH of each group reached the peak values of 7.53 (I-5), 7.55 (I-10), and 7.60 (I-20) on the 30th day, respectively. Interestingly, the pH finally was also kept at about 7.5, which was consistent with the results of previous studies. The Eh decreased significantly and reached the lowest value in the first 7 days, wherein the Eh of I-5, I-10, and I-20 groups were −267.67, −273, and −281 mV, respectively. The strongly reductive environment can promote the growth of reducing microorganisms and the stabilization of uranium in the remediation system. However, Eh showed a rising trend in I-5 and I-10 groups after 7 days, while Eh had no significant change in the I-20 group and remained below -200 mV. This is because the higher the inoculation volume, the more functional the microorganisms in the environmental system, and the more it is conducive to maintaining the reductive atmosphere.

#### Chemical Form Transformation During the Microbial Stabilization Process

**FIGURE 8:**
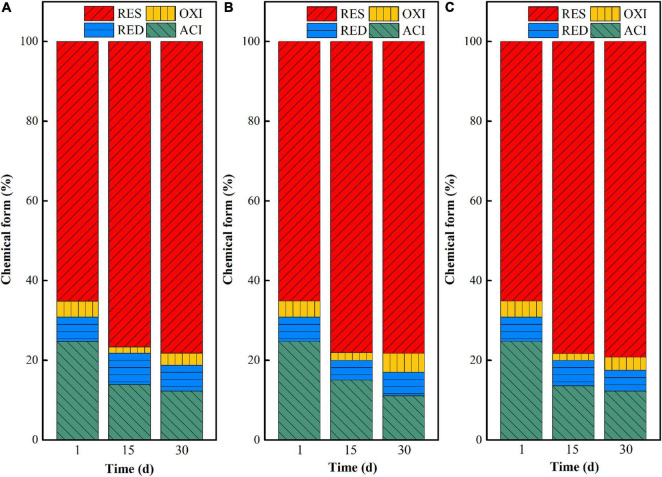
Chemical form changes of uranium in uranium tailings under different temperatures, **(A)** I-5; **(B)** I-10; and **(C)** I-20.

When the inoculation volumes were set as 5, 10, and 20%, the chemical form transformation of uranium after microbial stabilization for 1, 15, and 30 days was investigated, and the results are shown in Figure 8. On the 15th day, the acid-soluble uranium in I-5, I-10, and I-20 decreased by 10.85, 9.72, and 11.16%, while the residual uranium increased by 11.50, 12.96, and 13.18%, respectively. With the extension of time, the stabilization effect of uranium in the three groups all increased. Specifically, the decreases of uranium in the acid-soluble state were 12.46, 13.68, and 12.49% in I-5, I-10, and I-20, while the increases of uranium at the residual state were 13.10, 13.13, and 14.07%, respectively. These results indicated that the inoculation volume had a certain effect on the transformation of uranium in the microbial stabilization process. Overall, a high stabilization efficiency can be achieved in both I-10 and I-20 groups. However, besides the significant increase of residual uranium, the content of uranium at the acid-soluble state was decreased greatly at the same time in the I-10 group; that is, the comprehensive stabilization effect was better than those of the other two groups.

#### Microbial Community Response

As is shown in Table 4, there were significant differences in α diversity index under different inoculation volumes. In the I-5 group, the changes in the ACE and Chao 1 indexes basically showed a trend of decreasing first and then increasing, reaching the peak value on the 30th day (ACE-273.97, Chao 1-271.5); the Shannon index showed a steady upward trend and finally reached a peak of 3.51, while the Simpson index showed a stable declining trend during 1–15 days, and the lowest value obtained on the 15th and 30th days was 0.06. In the I-10 group, ACE and Chao 1 increased with the extension of time and finally reached the peak at day 30 (ACE-206.47, Chao 1-204.58); the Shannon and Simpson indexes fluctuated during the 1–30 days, and the peak/valley values were observed on the 15th day, which were 3.40 and 0.06, respectively. In the I-20 group, ACE and Chao 1 reached the peak on the 15th day, and their specific values were 145.32 and 141.5, respectively; the Shannon and Simpson indexes changed significantly versus time, with peak values of 2.52 and 0.15 on the 15th day, respectively. These results indicated that the inoculation volume was negatively correlated with the richness and diversity of the microbial community; that is, a higher inoculation volume was not conducive to the increase in microbial richness and diversity. This may be because the larger the inoculation volume, the more exogenous microorganisms are introduced, and the more intense the competition with endogenous microorganisms in the remediation process is, which causes the rapid consumption of nutrients in the environment and reduces the microbial richness and diversity in the system.

**TABLE 4:** Variation of α diversity index under different inoculation volume.

Group*	Shannon	Simpson	ACE	Chao 1	Coverage
I-5-1	0.80	0.54	114.31	82.07	0.999
I-5-3	1.90	0.20	119.10	72	0.999
I-5-7	1.92	0.25	65.98	66	0.999
I-5-15	3.27	0.06	190.19	184.23	0.999
I-5-30	3.51	0.06	273.97	271.5	0.999
I-10-1	1.57	0.25	57.90	61.75	0.999
I-10-3	1.22	0.50	68.49	55.87	0.999
I-10-7	2.10	0.25	98.80	83.14	0.999
I-10-15	3.40	0.06	179.81	185	0.999
I-10-30	2.90	0.18	206.47	204.58	0.999
I-20-1	1.60	0.28	107.20	102.565	0.999
I-20-3	1.33	0.37	59.07	58.17	0.999
I-20-7	2.20	0.18	105.53	80.5	0.999
I-20-15	2.52	0.15	145.33	141.5	0.999
I-20-30	2.28	0.24	140.96	141.21	0.999

*Remediation systems with different inoculation volumes were sampled at days 1, 3, 7, 15, and 30 and named according to inoculation volume and sampling time. For example, the 5 samples in I-5 were labeled I-5-1, I-5-3, I-5-7, I-5-15, and I-5-30, respectively.

**FIGURE 9:**
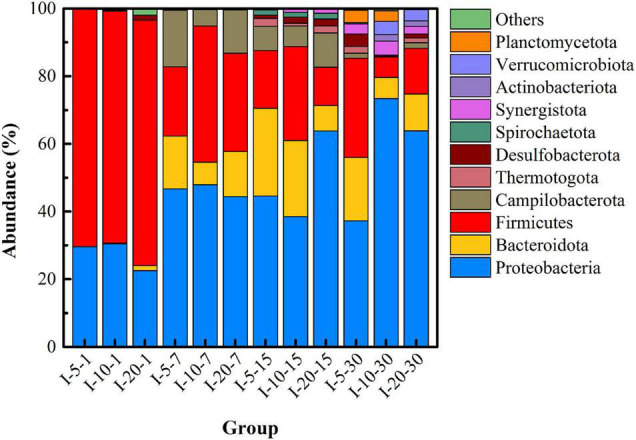
Microbial community structure at phylum level under different inoculation volume.

Figure 9 shows the microbial community structure (with abundance greater than 1%) at the phylum level under different inoculation volumes. In the initial stage of remediation (I-5-1, I-10-1, and I-20-1), the abundance of Desulfobacterota detected in I-20 was significantly higher than that in the other two groups. This indicated that a higher inoculation volume was beneficial to increase the richness of functional microorganisms in the initial stage. When the remediation lasted for 7 days, the abundance of Bacteroidota and Campilobacterota in the uranium tailings gradually increased. With the extension of time, microbial diversity increased significantly, but there was little difference in different groups (with different inoculation volumes) at the same period. Specifically, Firmicutes, Proteobacteria, Bacteroidota, Desulfobacterota, Synergustota, Thermotogota, and Actinobacteriota had always occupied the dominant position in the remediation system (the abundance was always greater than 1%). These results revealed that the inoculation volume significantly affected the richness and diversity of the microbial community at the initial stage, but the difference decreased with the extension of time.

The heatmap of the microbial community at the genus level (shown in Supplementary Figure 3) after remediation for 15 days was used to assist in analyzing the influence of inoculation volume on microbial community structure. On the whole, the main genera of microorganisms detected under different inoculation volumes included *Enterobacteriaceae*, *Sulfurospirillum*, *Proteiniphilum*, *Azospirillum*, *Macellibacteroides*, *Geobacter*, *Pseudomonas*, *Bacteroides*, and *Desulfosporosinus*. Specifically, the abundance of *Bact-08*, *Ruminiclostridium*, *Stenotrophomonas*, and *Azospira* in group I-5 was higher than that in other groups, indicating that low inoculation volume could promote the growth of endogenous microorganisms and enhance the diversity of endogenous microorganisms. This may be due to the small amount of exogenous functional microorganisms and the weak competition to endogenous microorganisms. Meanwhile, it was found that the abundance of sulfate-reducing and iron-reducing bacteria in the I-10 and I-20 groups was much higher than that in the I-5 group. In particular, the combined abundance of *Azospirillum*, *Geobacter*, *Desulfosporosinus*, and *Sedimentibacter* was 10.74 and 20.28% in I-10 and I-20, respectively. In addition, the generic biodiversity in group I-20 was significantly lower than that in the other two groups, which was consistent with the change in α diversity index as shown in Table 4.

Unfortunately, PSB did not become the dominant microorganism in all the high-throughput sequencing results; that is, they were not shown in the microbial community with an abundance greater than 1% at the phylum level (Figures 3, 6, 9) and at the top 30 genus level (Supplementary Figures 1–3). This may be due to the poor tolerance of *Pantoea* sp. grinm-12 in the uranium-contaminated environment, so they were in a disadvantage position in competition with other endogenous microorganisms; eventually, their activity was reduced (even died) in the uranium tailings. Considering the chemical form transformation of uranium under different inoculation volumes as shown in Figure 8, an inoculation volume that could combine high stabilization efficiency with an acceptable level of microbial richness and diversity should be selected, that is, 10% inoculum was recommended to be considered for the practical stabilization of uranium tailings.

### Ordination Analysis Based on Environmental Conditions

#### Principle Coordinate Analysis Under Different Conditions

**FIGURE 10:**
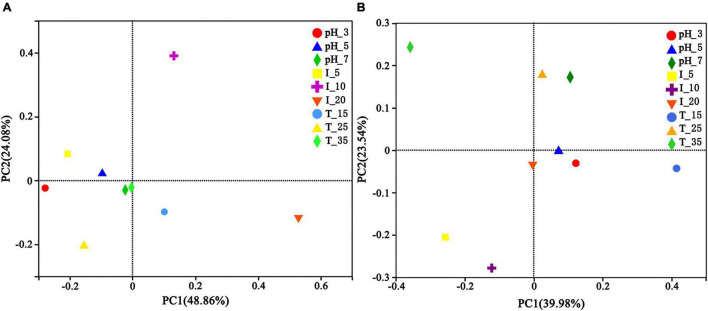
Principle coordinates analysis (PCoA) of uranium tailings after stabilization for **(A)** 1 day and **(B)** 15 days under different environmental conditions.

Principle coordinate analysis (PCoA) was used to analyze the uranium tailings after microbial stabilization for 1 and 15 days to understand the effect of different environmental conditions on microbial community composition. As is shown in Figure 10A, environmental conditions had little effect on the microbial community composition in the initial remediation stage, and the microbial community structures were consistent under different pH and temperatures. However, the difference in microbial community composition between I-10 and I-20 groups was greater than that of other groups. It has been reported that increasing the initial biomass concentration can improve the removal rate of uranium but will lead to an intensified competition of microbial growth at the same time (Chabalala and Chirwa, 2010). With the extension of time, the microbial community structure changed significantly under different environmental conditions and then evolved into a relatively stable functional microbial community. As is shown in Figure 10B, the contribution rates of PC1 and PC2 were 39.98 and 23.54%, respectively, and the cumulative contribution rate of PC1 and PC2 was 63.52%. In particular, temperature and inoculum volume had obvious effects on microbial community structure succession during the remediation process. The sample distance was the largest in groups under different temperatures, followed by inoculation volume, and the sample distance was the smallest in groups under different pH. Therefore, it can be concluded that different environmental conditions can affect and change the composition of the microbial community, wherein temperature and inoculation volume are relatively more influential factors.

#### Canonical Correlation Analysis Under Different Conditions

**FIGURE 11:**
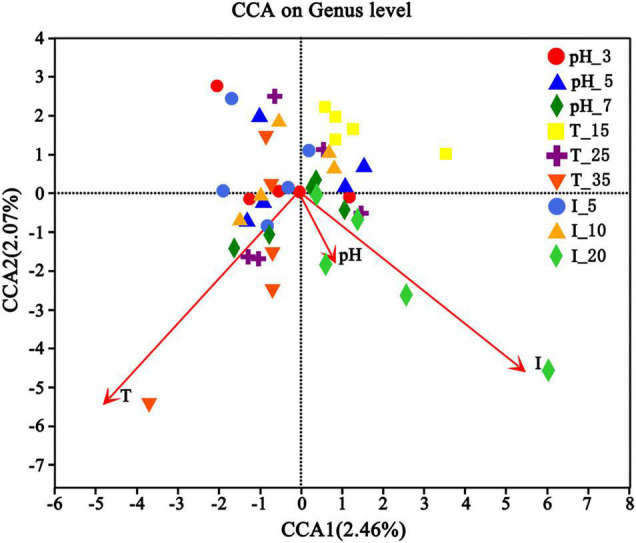
Canonical correlation analysis (CCA) of uranium tailings after stabilization for 15 days under different environmental conditions.

Different environmental factors can significantly change the microbial community structure (Liu, 2019). In order to explore whether the microbial community structure is related to environmental factors, the canonical correlation analysis (CCA) test was conducted according to the gene sequences of high-throughput sequencing and specific conditions. The main environmental factors were temperature (15, 25, and 35°C), inoculation volume (5, 10, and 20%), and pH (3, 5, and 7). It is shown in Figure 11 that environmental factors definitely had different contributions to the microbial communities, among which temperature and inoculation volume showed more significant influence. Overall, the influence of environmental factors on microbial community structure followed this order: inoculation volume > temperature > pH.

## Conclusion

In this study, the effects of temperature, pH, and inoculation volume on the microbial stabilization efficiency of uranium in uranium tailings were investigated by mixed SRB-PSB, and high-throughput sequencing was used to assist in revealing the microbial community response. The results showed that the optimal pH of the remediation system was 5, which can effectively reduce the Eh of the environmental system, stabilize the pH of culture system, and promote the transformation of acid-soluble uranium to residual uranium. Combined with microbial analysis, it was found that pH = 5 could significantly increase the microbial richness and diversity of the remediation system, effectively maintain the microbial reduction system, and significantly change the microbial community structure. Then, 25°C was determined as the optimal temperature for the SRB-PSB coculture system to interact with uranium; that is, the stabilization process can be carried out at room temperature. Microbiological analysis showed that the microbial richness and diversity could be significantly increased at 25°C. At the same time, the abundance of some functional microorganisms, such as *Desulfurispora*, *Desulfofarcimen*, *Desulfovibrio*, and *Geobacter*, were relatively high, which are beneficial to promote the stabilization of uranium. In addition, the highly efficient stabilization of uranium and the stable succession of the microbial community structure could be achieved simultaneously when the inoculation volume was about 10%. In particular, the abundance of functional microorganisms such as *Azospirillum*, *Geobacter*, *Desulfosporosinus*, and *Sedimentibacter* can be maintained at a high level. Finally, PCoA and CCA analyses showed that there were significant differences in the effects of temperature, pH, and inoculation volume on microbial community structure, and the influence sequencing was as follows: inoculation volume > temperature > pH. This study puts forward the optimal environmental conditions for microbial stabilization of uranium in uranium tailings by mixed SRB-PSB and reveals the reconstruction and response process of the microbial community under different environmental conditions, which will provide data reference for the construction of *in situ* stabilization theory of uranium-contaminated sites.

## Data Availability Statement

The original contributions presented in the study are included in the article/Supplementary Material, further inquiries can be directed to the corresponding author.

## Author Contributions

XL provided the idea of this work. CT and YL performed the experiment and detected and analyzed the data. YL prepared the figures and wrote the manuscript. XH detected physiochemical properties. SC and XZ were involved in experimental design. CT and XZ collected the samples. MZ and BC revised the manuscript. All authors contributed to the article and approved the submitted version.

## Conflict of Interest

XL, MZ, and BC were employed by GRIMAT Engineering Institute Co., Ltd. The remaining authors declare that the research was conducted in the absence of any commercial or financial relationships that could be construed as a potential conflict of interest.

## Publisher’s Note

All claims expressed in this article are solely those of the authors and do not necessarily represent those of their affiliated organizations, or those of the publisher, the editors and the reviewers. Any product that may be evaluated in this article, or claim that may be made by its manufacturer, is not guaranteed or endorsed by the publisher.
