# Supplementary material for: Optimization of Environmental Conditions for Microbial Stabilization of Uranium Tailings, and the Microbial Community Response
Source: Front Microbiol. 2021 Dec 13;12:770206. doi: 10.3389/fmicb.2021.770206 (PMC8710664; doi:10.3389/fmicb.2021.770206)
Supplement: Supplementary file 1 [file Data_Sheet_1.docx]

**Additional files**


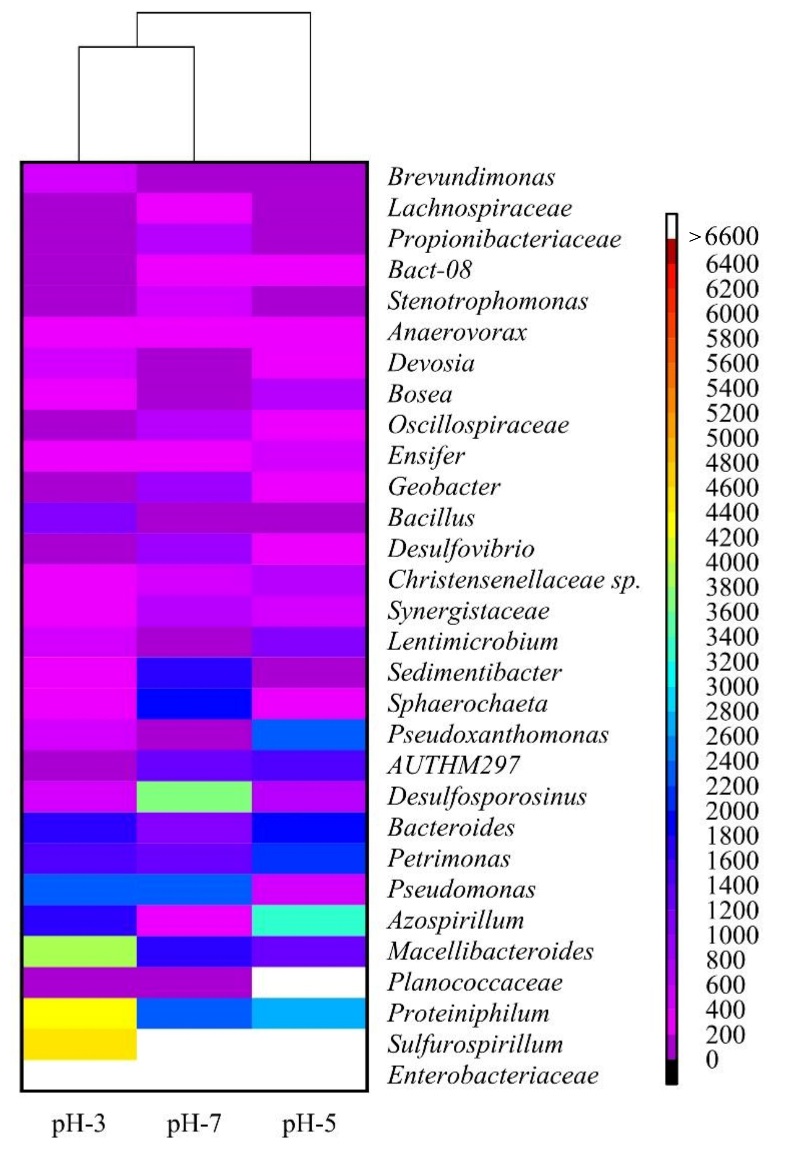


Fig. S1 Heatmap analysis of microbial community at the genus level under different pH.


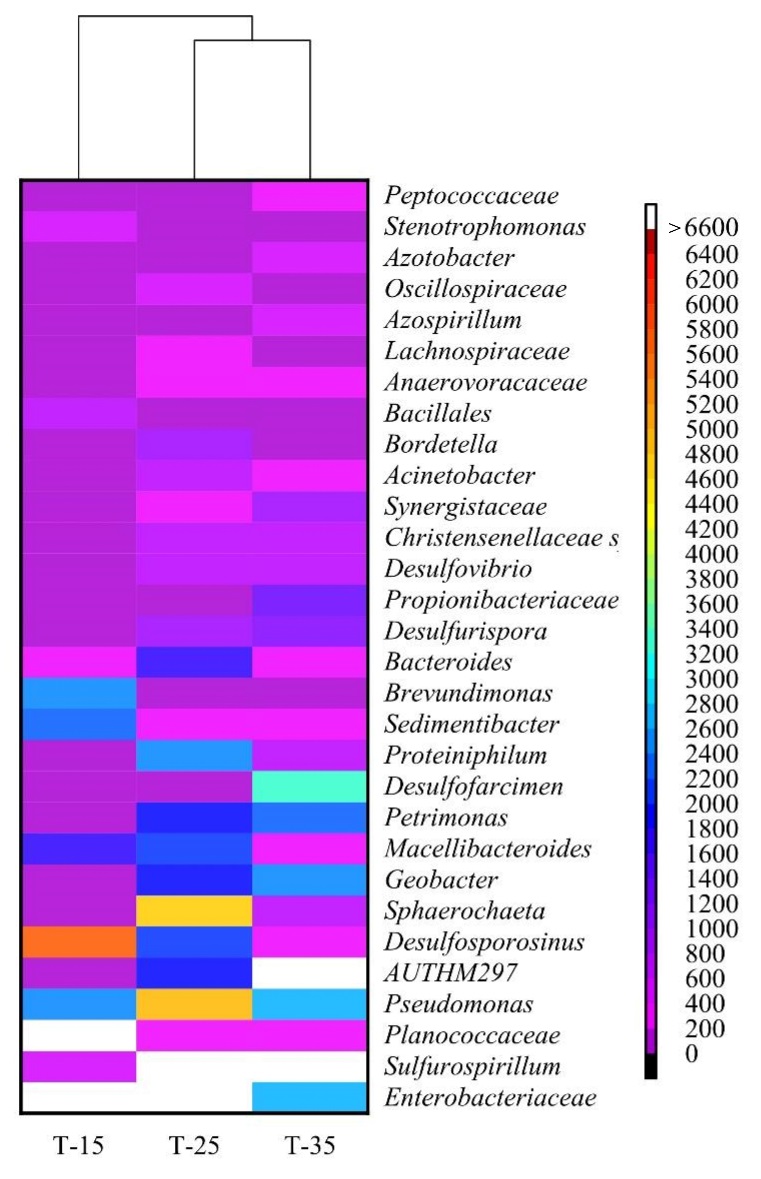


Fig. S2 Heatmap analysis of microbial community at the genus level under different temperature.


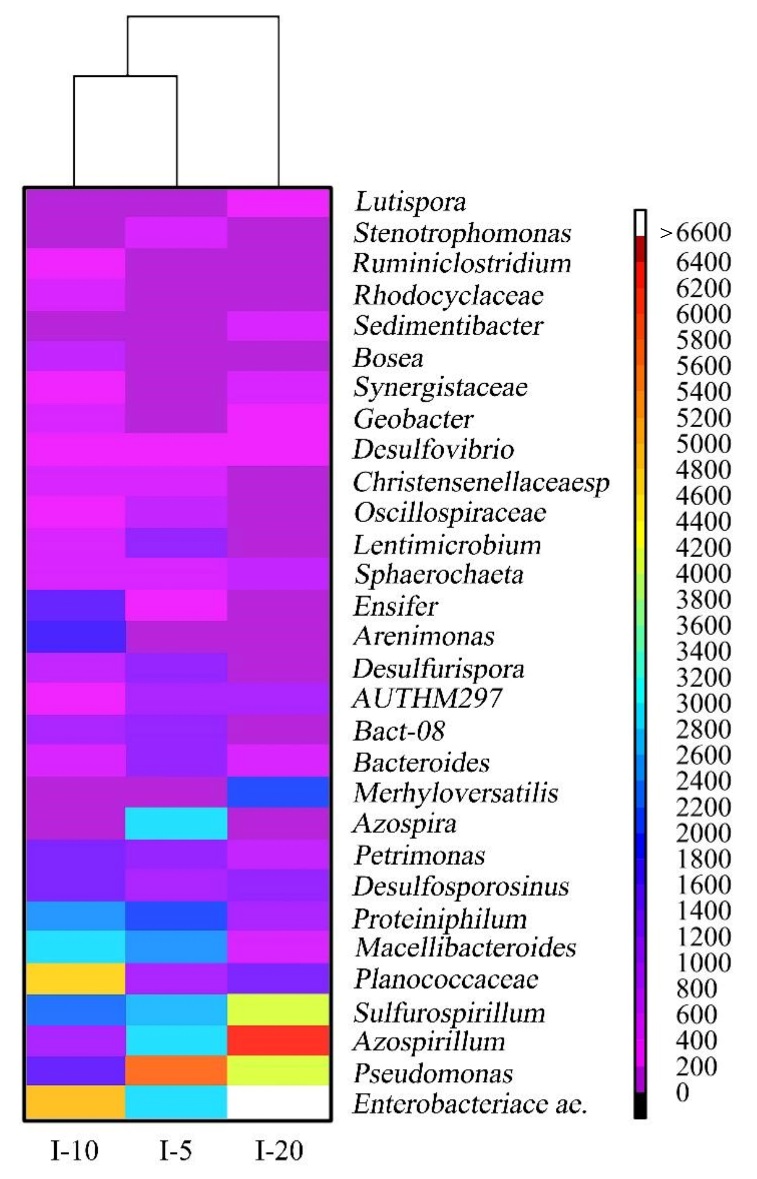


Fig. S3 Heatmap analysis of microbial community at the genus level under different inoculation volume.
